# Phytolacca Decandra in Orchitis

**Published:** 1885-08

**Authors:** W. O’Daniel

**Affiliations:** Bullard’s, Ga.


### PHYTOLACCA DECANDRA IN ORCHITIS.

By W. O'DANIEL, M. D., BULLARD'S, GA.

No doubt most physicians have experienced trouble and anxiety in giving patients, suffering from inflammation of the testicle, no matter whether gonorrhœal or not, the expected speedy relief. For several years past I have adopted the following plan of treatment: If there is much inflammation and swelling of the affected parts, I usually have the patient to stand on his feet, which increases the already turgescent condition of the scrotal veins, when I puncture them, thereby relieving, to some extent, the abnormal congestion by the free escape of blood from the distended veins.

After which I advise patient to remain in bed and take from four to six drops of the fluid extract of phytolacca decandra every three or four hours, until the specific effect of the drug is at least partially had. I then lessen the dose or lengthen the interval, according to circumstances. This, with an ointment of belladonna and phytolacca, applied to the swollen and inflamed parts, with an anodyne, if necessary, to relieve pain and promote rest, and a well-fitting suspensary bandage after convalescence, constitutes the most satisfactory treatment known to me.

